# [^123^I]FP-CIT ENC-DAT normal database: the impact of the reconstruction and quantification methods

**DOI:** 10.1186/s40658-017-0175-6

**Published:** 2017-01-28

**Authors:** Livia Tossici-Bolt, John C. Dickson, Terez Sera, Jan Booij, Susanne Asenbaun-Nan, Maria C. Bagnara, Thierry Vander Borght, Cathrine Jonsson, Robin de Nijs, Swen Hesse, Pierre M. Koulibaly, Umit O. Akdemir, Michel Koole, Klaus Tatsch, Andrea Varrone

**Affiliations:** 1 Department of Medical Physics, University Hospital Southampton NHS Foundation Trust, Southampton, UK; 2 Institute of Nuclear Medicine, University College London Hospital NHS Foundation Trust, London, UK; 3 Department of Nuclear Medicine and Euromedic Szeged, University of Szeged, Szeged, Hungary; 4 Department of Nuclear Medicine, Academic Medical Center, University of Amsterdam, Amsterdam, The Netherlands; 5 Department of Nuclear Medicine, Medical University of Vienna, Vienna, Austria; 6 Medical Physics Unit, Az. Ospedaliera Universitaria San Martino, Genoa, Italy; 7 Nuclear Medicine Division, Mont-Godinne Medical Center, Université Catholique de Louvain, Yvoir, Belgium; 8 Department of Nuclear Medicine, Medical Physics, Karolinska University Hospital, Stockholm, Sweden; 9 Neurobiology Research Unit and Department of Clinical Physiology, Nuclear Medicine and PET, Copenhagen University Hospital, Rigshospitalet, Copenhagen, Denmark; 10 Department of Nuclear Medicine, University of Leipzig, Leipzig, Germany; 11 Nuclear Medicine Department, Centre Antoine Lacassagne, University of Nice-Sophia Antipolis, Nice, France; 12 Department of Nuclear Medicine, Faculty of Medicine, Gazi University, Ankara, Turkey; 13 Nuclear Medicine, University Hospital and K.U. Leuven, Leuven, Belgium; 14 Department of Nuclear Medicine, Municipal Hospital of Karlsruhe Inc., Karlsruhe, Germany; 15 Department of Clinical Neuroscience, Center for Psychiatric Research, Karolinska Institutet, Stockholm, Sweden

**Keywords:** ^123^I, FP-CIT, SPECT, Quantification, Reconstruction, Calibration, Specific binding ratio

## Abstract

**Background:**

[^123^I]FP-CIT is a well-established radiotracer for the diagnosis of dopaminergic degenerative disorders. The European Normal Control Database of DaTSCAN (ENC-DAT) of healthy controls has provided age and gender-specific reference values for the [^123^I]FP-CIT specific binding ratio (SBR) under optimised protocols for image acquisition and processing. Simpler reconstruction methods, however, are in use in many hospitals, often without implementation of attenuation and scatter corrections. This study investigates the impact on the reference values of simpler approaches using two quantifications methods, BRASS and Southampton, and explores the performance of the striatal phantom calibration in their harmonisation.

**Results:**

BRASS and Southampton databases comprising 123 ENC-DAT subjects, from gamma cameras with parallel collimators, were reconstructed using filtered back projection (FBP) and iterative reconstruction OSEM without corrections (IRNC) and compared against the recommended OSEM with corrections for attenuation and scatter and septal penetration (ACSC), before and after applying phantom calibration. Differences between databases were quantified using the percentage difference of their SBR in the dopamine transporter-rich striatum, with their significance determined by the paired t test with Bonferroni correction.

Attenuation and scatter losses, measured from the percentage difference between IRNC and ACSC databases, were of the order of 47% for both BRASS and Southampton quantifications. Phantom corrections were able to recover most of these losses, but the SBRs remained significantly lower than the “true” values (*p* < 0.001). Calibration provided, in fact, “first order” camera-dependent corrections, but could not include “second order” subject-dependent effects, such as septal penetration from extra-cranial activity. As for the ACSC databases, phantom calibration was instrumental in compensating for partial volume losses in BRASS (~67%, *p* < 0.001), while for the Southampton method, inherently free from them, it brought no significant changes and solely corrected for residual inter-camera variability (−0.2%, *p* = 0.44).

**Conclusions:**

The ENC-DAT reference values are significantly dependent on the reconstruction and quantification methods and phantom calibration, while reducing the major part of their differences, is unable to fully harmonize them. Clinical use of any normal database, therefore, requires consistency with the processing methodology. Caution must be exercised when comparing data from different centres, recognising that the SBR may represent an “index” rather than a “true” value.

## Background

[^123^I]FP-CIT is a well-validated radiopharmaceutical that binds to the dopamine transporter, which is intensively expressed in the striatum, and is used in clinical practice to support the diagnosis of dopaminergic degenerative movement disorders like Parkinson’s disease. The EANM Research Ltd (EARL) “ENC-DAT” project (European Normal Control Database of DaTSCAN) has provided a multicenter database of [^123^I]FP-CIT SPECT scans acquired from European healthy controls, which constitutes an invaluable reference for the quantification of [^123^I]FP-CIT clinical studies [1]. Its optimal use requires adherence to standardized acquisition and reconstruction protocols [2]. In particular, OSEM reconstruction with corrections for attenuation and scatter and septal penetration has been recommended as the most accurate approach, in conjunction with a preliminary phantom calibration of the gamma camera [3]. Many hospitals, however, opt for simpler reconstructions and the use of filtered back projection (FBP), often without any corrections, is still widespread. Quantification methods of the striatal specific binding ratio (SBR), obtained with [^123^I]FP-CIT SPECT, also vary considerably across vendors and bespoke algorithms. Moreover, the use of phantom calibration is still infrequent.

**Fig. 1:**
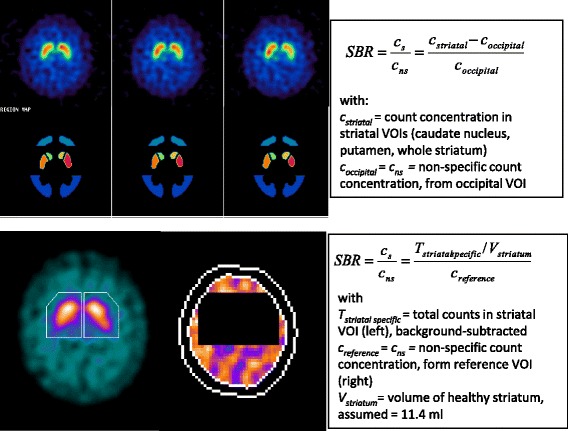
The two methods used for measuring the striatal specific binding ratio (SBR), defined as the ratio of specific to non-specific striatal count concentrations, SBR = *c*_*s*_ */c*_*ns*_. *Top*: BRASS quantification method [4]. *c*_*s*_ and *c*_*ns*_ are measured from count concentrations using anatomical VOIs for the sub-striatal structures (caudate and putamen) and the occipital lobes, respectively. The striatal SBR used in this work was obtained by dividing the total counts from these two VOIs by their combined volume. The small volumes of these structures render these concentration measurements susceptible to partial volume losses. *Bottom*: Southampton quantification method [5]. *c*_*s*_ is derived from a measure of total counts in a geometrical VOI for the striatum. The generous dimensions of this VOI ensure that all counts related to striatal binding are captured, including those detected outside the anatomical boundary, thus averting under-estimations due to partial volume losses. *c*_*ns*_ is also measured from a large VOI, encompassing the whole cortex with the exception of the striata and excluding the outer rim beset by peripheral partial volume losses

The primary aim of this work is to investigate the impact of the reconstruction, quantification method and phantom calibration on the ENC-DAT database. Databases derived with FBP and OSEM without any corrections have been compared to the recommended reconstruction with and without phantom calibration. Their quantification has been carried out with two different methods, BRASS (Hermes Medical Solutions) [4] and the Southampton method [5], which, with their fundamentally different approach to partial volume losses (Fig. 1), provide an invaluable resource to assess its impact on the SBR value.

Finally, the availability of all these data, the SBRs from three different reconstructions and two quantification methods, provides an ideal platform to extensively test the value of the phantom calibration and its ability to compensate for any of the three SPECT limitations, attenuation, scatter/septal penetration and partial volume. As a secondary aim of this work, the calibration performance in restoring the “true” SBR values, and thus harmonising different databases, will be investigated and its limitations addressed.

## Methods

A total of 48 scans of the same striatal phantoms and 123 healthy volunteers from nine of the “ENC-DAT” cameras with parallel collimators, were reconstructed in three different ways, with filtered back projection (FBP) and ordered subsets expectation maximisation (OSEM) without corrections, FBP and IRNC, and with OSEM with corrections for attenuation and scatter and septal penetration, ACSC. The healthy controls covered an age range between 30 and 90 years and were similarly gender balanced in term of numbers and average age [1].

The scans of the anthropomorphic striatal phantom (Radiology Support Devices Inc., Long Beach, CA, USA) with known filling ratios (striatum versus background) ranging from 10:1 to 1:1 (uniform filling), provided the camera-specific calibration factor needed to harmonize differences in camera performance and recover the true filling ratio [3].

The three reconstructions were performed on two platforms, Xeleris (GE Healthcare) for the BRASS analysis and MAPS (Link Medical) for the Southampton analysis. Both platforms used the original OSEM algorithm [6], with 10 iterations and 10 subsets (for 120 projections) or 8 iterations and 12 subsets (for 128 projections). Post-filtering with Butterworth cutoff = 0.55 (Xeleris) and 0.5 (Link) cycles/cm and order 10 was applied for all OSEM reconstructions, while for FBP pre-filtering with Butterworth cutoff of 0.55 cycles/cm and order 10 was applied to the raw projections. Attenuation correction for the human controls was based on a variable ellipsoid map that followed the contour of the head and filled with a uniform attenuation coefficient mu = 0.143 cm^−1^ [1, 3]. The contour was automatically defined using thresholding, which was based on the maximum voxel counts on Xeleris and on the average background on Link. As for the phantom, a similar approach was adopted on Xeleris, while a standard ellipsoid of fixed size (140 × 190 mm) corresponding to the maximum dimensions of the outer skull was used on Link in order to account for its lack of radioactivity [3]. The correction for scatter and septal penetration was based on the triple energy window (TEW) method, using satellite windows acquired below and above the photopeak; more details can be found in [3].

The SBR was measured using two different methods, BRASS (version 3.5, Hermes Medical Solutions) [4] and the Southampton methods [5], which are representative of two widespread and fundamentally different approaches to SBR quantification. The first, BRASS, is based on registration of the images to a [^123^I]FP-CIT template and on direct measurement of striatal count-concentration from template volumes of anatomical shape. Conversely, the Southampton method operates directly on the original data and derives the striatal count-concentration from a measure of total counts, according to the “specific uptake size index” approach for overcoming the partial volume effect [7]; a value of 11.4 ml was used for the striatal volume [3] (Fig. 1).

Dedicated phantom calibration was applied to each of the six databases, with recovery coefficient specific to the three reconstructions considered, FBP, IRNC, and ACSC, and to the two quantification methods, BRASS and Southampton. The ability of the calibration to compensate for attenuation, scatter and partial volume losses, which is the foundation of the harmonisation process, was tested by comparison of databases before and after its application.

Linear regression analysis was applied to each database to characterize the age decline observed in normal subjects; the standard error of the regression was used to define the 95% CI limits of the measured SBRs. The regression lines allowed the derivation of age-corrected SBRs (referenced to an age of 65 years), whose means, standard deviations (SD) and coefficient of variation (CoV) provided a concise characterisation of the six databases. The value of 65 years, chosen as reference age, was considered to be representative of the average age of the patient seen in clinical practice; in particular it coincides with the average age of the patients included in the work of Dickson et al., where the clinical relevance of the six databases considered in this study is explored [8].

In depth comparisons between databases were done using the % difference:

1 $$ \%\mathrm{difference}=100\ast \frac{1}{2 N}{\displaystyle {\sum}_i\frac{A_i-{B}_i}{\left({A}_i+{B}_i\right)/2}}\kern0.5em \mathrm{i}=1,2 N\left(\mathrm{N}=\mathrm{numberofhumancontrols}\right), $$

where *A* and *B* represent any two databases, either from different reconstructions and/or quantification methods, before and/or after phantom calibration. The average is done over 2 N values because of the separate contributions of right and left striata for each of the *N* subjects.

The significance of their differences was tested with the paired *t* test with Bonferroni correction for multiple comparisons (significance level *α* = 0.05/*m*, with *m* = number of multiple hypothesis tested).

The choice of *A* and *B* in Eq.1 was related to the specific quantitative aspect addressed. For example, the effect of the attenuation and scatter losses was investigated by selecting *A* = IRNC vs *B* = ACSC (both pre-calibration databases, keeping the quantification method constant); the effect of partial volume using *A* = BRASS and *B* = Southampton (both pre-calibration, keeping the reconstruction method constant); the performance of the phantom calibration using *A* = before and *B* = after (keeping both reconstruction and quantification constant).

Phantom calibration provides a methodology for reducing inter-camera variations and recovering the true SBR value in human studies; however, its success is ultimately linked to the extent to which it is representative of the clinical context. This point was addressed by investigating the relevance of extra-brain activity which is not accounted for in a phantom study. The high uptake and retention of [^123^I]FP-CIT in lungs, liver, and intestines and sometimes in salivary glands and thyroid [9] is, in fact, expected to contribute to the brain image, particularly through septal penetration from the ^123^I high-energy gamma emissions [10, 11]. The magnitude of this contribution was indirectly estimated from a comparison of the phantom (brain activity only) and the human (brain and extra-brain activity) data for each camera. For this purpose, the percentage of the total counts in the raw-projections in the lower and upper scatter windows (SC_l_ and SC_u_) relatively to the photopeak counts (PH) was calculated as:

2 $$ \%\mathrm{S}\mathrm{C}=100*\frac{\mathrm{total}\ \mathrm{counts}\ \mathrm{S}\mathrm{C}\ /\ {W}_{\mathrm{SC}}}{\mathrm{total}\ \mathrm{counts}\ \mathrm{PH}\ /\ {W}_{\mathrm{PH}}}\mathrm{with}\ \mathrm{S}\mathrm{C}=\mathrm{S}{\mathrm{C}}_{\mathrm{l}}\kern0.5em \mathrm{or}\kern0.5em \mathrm{S}{\mathrm{C}}_{\mathrm{u}} $$

where *W*_PH_ and *W*_SC_ represent the widths of the photopeak and scatter windows (SC_l_ and SC_u_), respectively.

For each camera, the average values of the % SC, obtained for the phantom and for the human data, were then compared using their percentage difference:

3 $$ \%\kern0.5em \mathrm{extra}\hbox{-} \mathrm{brain}\ \mathrm{contribution}\ \mathrm{t}\mathrm{o}\ \mathrm{S}\mathrm{C}=100*\frac{\mathrm{average}\left(\%{\mathrm{SC}}_{\mathrm{controls}}\right)\hbox{-} \mathrm{average}\left(\%\kern0.3em {\mathrm{SC}}_{\mathrm{phantom}}\right)}{\left[\mathrm{average}\left({\%\mathrm{SC}}_{\mathrm{controls}}\right)+\mathrm{average}\left({\%\mathrm{SC}}_{\mathrm{phantom}}\right)\right]/2} $$

This subtraction, of the scatter (SC) contribution due to brain only (phantom) from the total scatter (controls), provided an indirect estimate of the scatter contribution due to extra-brain activity for each camera.

**Fig. 2:**
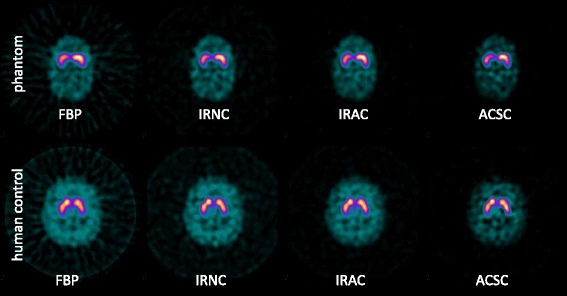
Examples of FBP and iterative reconstructions for a phantom study with highest filling ratio (Left=10:1, Right=8:1,*top row*) and a human control (*bottom row*), both acquired on an Infinia Hawkeye camera and reconstructed on the Link Medical workstation. Each image represents one (1 pixel-thick) central slice and is normalised to its own maximum

Furthermore, to further characterize the relevance of extra-brain activity, phantom and human data were also compared in terms of the % difference of their respective IRNC and ACSC databases. In order to separate the individual magnitude of the AC and SC losses, a further reconstruction was considered, IRAC (OSEM with attenuation correction only). This comparison was carried out using the Southampton method, to eliminate the confounding effect of partial volume losses present in BRASS, and it was also limited to phantom fillings representative of the healthy human striatal SBR for [^123^I]FP-CIT SPECT studies (that is 10:1, 8:1, 5:1, and 4:1). Note that the use of the IRAC reconstruction in this paper is limited to this comparison only. Exemplary reconstructions for a phantom (higher filling) and a human control study are shown in Fig. 2.

## Results

### ENC-DAT databases

**Fig. 3:**
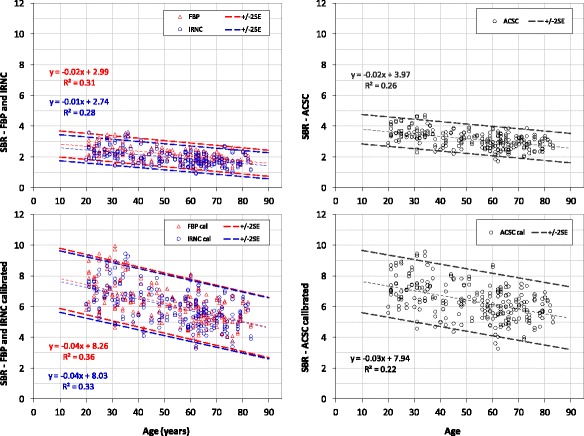
ENC-DAT database of normal controls, BRASS quantification. Striatal specific binding ratios (SBR) vs age derived from various reconstructions: FBP (*red*), IRNC (blue) and ACSC (*black*), before (*top row*) and after (*bottom row*) phantom calibration. Their respective linear fit and the 95% CI (two standard error of the regression) are also shown following the same colour code

**Fig. 4:**
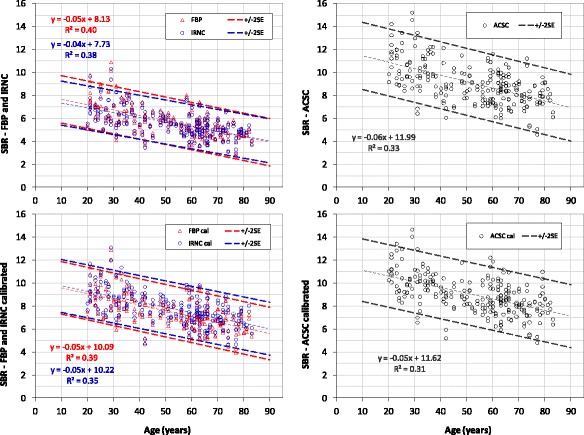
ENC-DAT database of normal controls, Southampton quantification. Striatal specific binding ratios (SBR) vs age derived from various reconstructions, following the same conventions as in Fig. 2: FBP (*red*), IRNC (*blue*) and ACSC (*black*), before (*top row*), and after (*bottom row*) phantom calibration. Note the wider *y*-axis range compared to Fig. 2

The FBP, IRNC, and ACSC control databases quantified using BRASS and Southampton methods are shown in Figs. 3 and 4, respectively. The striatal SBR is plotted as a function of age. The graphs on the left correspond to reconstructions without any corrections (FBP and IRNC), those on the right to OSEM with corrections for attenuation and scatter and septal penetration (ACSC). The graphs on the top row represent the direct results of the SBR measurements, those on the bottom are derived from them by applying phantom calibration.

Displayed on each graph are also the line of best fit, which describes the age decline to be expected in a healthy population, and the 95% CI limits calculated from the standard error of the regression. The age-corrected mean values, SD and CoV of these databases, with SBRs referenced to the age of 65 years using the regression lines, have been summarised in Table 1.

**Table 1:** Means, standard deviations and coefficient of variations of the age-corrected striatal specific binding ratio (SBR) of all controls for the six databases explored in this study. The reference age is of 65 years

Average SBRs (SD) *CoV*	FBP	IRNC	ACSC	FBP cal	IRNC cal	ACSC cal
BRASS	1.98 (0.42) *21%*	1.78 (0.42) *24%*	2.96 (0.48) *16%*	5.65 (0.98) *17%*	5.55 (1.00) *18%*	5.99 (1.02) *17%*
Southampton	5.11 (1.03) *20%*	5.06 (0.96) *19%*	8.33 (1.46) *17%*	6.85 (1.14) *17%*	7.20 (1.15) *16%*	8.38 (1.37) *16%*

The reconstructions with no corrections (FBP and IRNC) display a small but significant difference for both quantification methods (*p* < 0.001). On average, FBP gives higher SBRs, an indication that, despite the high number if iterations used for the IRNC, the contrast of FBP remains marginally superior. Furthermore, the non-negativity constraint of OSEM could also contribute to a reduction of the striatal SBR, as it is known to possibly lead to a positive bias in noisy low-counts regions such as those of the cortical background in the [^123^I]FP-CIT SPECT images where the expression of dopamine transporter is low [2].

ACSC corrections, as expected, bring a significant increase in the SBR values, ~47%, for both quantification methods (Table 2). Calibration helps to reduce significantly the difference between the ACSC and the FBP/IRNC databases, but does not eliminate it completely. The post-calibration FBP/IRNC values consistently under-estimate the corresponding ACSC ones across all 2 N terms of Eq. 1. The overall residual difference, ~6% for BRASS and ~15% for Southampton, is statistically significant in both cases (*p* < 0.001; Bonferroni corrected significance level *α* = 0.05/4 = 0.0125).

**Table 2:** Impact of ACSC corrections on the control databases

SBR % difference: reconstruction method	BRASS	Southampton
ACSC-IRNC pre calibration *(impact of ACSC losses)*	47.4	47.7
ACSC-IRNC post calibration *(expected = 0)*	5.8	14.5

The top row shows that attenuation and scatter and septal penetration losses are practically identical for the two quantification methods, as expected. Phantom calibration (bottom row) is unable to fully recover them for the IRNC databases and their difference with the ACSC ones remains significant for both methods (*p* < 0.001)

*SBR* striatal specific binding ratio

These findings raise the question on how well the phantom, on which the calibration is based, is representative of a human study. In particular, differences with respect to scatter and septal penetration are likely to be present, due to the activity distribution in the rest of the human body. This point has been addressed in the next section.

The biggest difference across databases, however, is associated with the quantification method. The comparison of Figs. 3 and 4 shows that BRASS produces SBRs systematically lower than that of the Southampton method for any of the reconstructions considered, with a percentage difference of ~96% (Table 3, pre-calibration). This difference, ultimately representative of the magnitude of the partial volume losses, cannot be fully compensated by phantom calibration (Table 3, post-calibration). The calibrated BRASS values, in fact, remain consistently lower than the Southampton ones across all the 2 N terms of Eq. 1, resulting in a percentage difference between these two methods ~26% for IRNC and 34% for ACSC. Accordingly, the averages of these two groups are found to be significantly different (*p* < 0.001, Bonferroni corrected significance level *α* = 0.05/4 = 0.0125).

**Table 3:** Impact of quantification method

SBR % difference quantification method	Southampton—BRASS	
	IRNC	ACSC
Pre calibration *(impact of PVE)*	96.0	95.9
Post calibration *(expected = 0)*	25.7	33.9

The difference of the un-calibrated Southampton and BRASS databases (first row) represents the magnitude of partial volume losses in human studies. The limitation of phantom calibration in compensating for this effect is reflected in the residual differences of the post-calibration databases

*SBR* striatal specific binding ratio

Finally, Table 4 summarises the extent of the change brought on each database by phantom calibration. This varies greatly from a maximum for the NC BRASS database to a minimum for the ACSC Southampton reconstruction. For the latter, the *t* tests confirms that there is no statistically significant difference between the Southampton ACSC pre- and post-calibration averages (*p* = 0.44, Bonferroni corrected significance level *α* = 0.05/6 = 0.008).

**Table 4:** Impact of phantom calibration, as given by the % difference of each database before and after calibration

SBR % difference: phantom calibration	BRASS	Southampton
FBP (post-pre)	96.0	27.8
IRNC (post-pre)	102.1	33.6
ACSC (post-pre)	67.4	−0.2

With ACSC, this difference represents the phantom recovery of partial volume losses, which is significant for BRASS but not for the Southampton method (*p* = 0.44)

*SBR* striatal specific binding ratio

### Limitations of phantom calibration

The results for the comparison of phantom and human data, aimed to evaluate the relevance of extra-brain activity in the rest of the body, are summarised in Tables 5 and 6.

**Table 5:** Impact of extra-brain activity: comparison of scatter and photopeak counts in human and phantom raw projections

Camera	Phantom		Human controls		% difference 100*(controls-phantom)/average	
	SCl/PH (%)	SCu/PH (%)	SCl/PH (%)	SCu/PH (%)	% Diff for SCl	% Diff for SCu
GE INFINIA2	70.1	53.9	72.9	56.9	3.9	5.4
GE INFINIA1	67.6	56.3	68.9	65.8	1.8	15.7
Philips IRIX (MEGP)	63.5	37.5	61.4	40.3	-3.4	7.2
GE MILLENNIUM	60.1	49.0	65.0	58.4	7.7	17.4
Siemens SYMBIA1	58.1	48.7	61.4	50.3	5.5	3.3
Siemens SYMBIA2	59.3	47.2	57.4	53.9	-3.2	13.2
Siemens ECAM1	59.2	46.1	57.1	49.4	-3.5	6.9
Siemens ECAM2	61.5	48.3	58.6	52.4	-4.9	8.2
Siemens ECAM3	61.6	48.7	63.1	55.1	2.3	12.2
Mediso NUCLINE	66.7	41.3	67.5	51.3	1.3	21.6
Trionix TRIAD1	58.9	41.5	62.0	52.0	5.2	22.6
Trionix TRIAD2	63.3	54.0	67.0	66.3	5.7	20.4

Columns 2-5: total counts in SCl and SCu windows are expressed relatively to photopeak (% of PH) counts. In the last two columns (6, 7), the results for human and phantom data are compared in terms of their percentage difference

**Table 6:** Impact of ACSC corrections (combined and separate contributions) in phantom and human studies: percentage differences of the SBR measured with the Southampton method from the various reconstructions

SBR % difference contributions of AC and SC	Southampton method	
	Phantom	Controls
ACSC-IRNC *(impact of ACSC corrections combined)*	39.4	47.7
IRAC-IRNC *(impact of AC losses alone)*	15.5	16.5
ACSC-IRAC *(impact of SC losses alone)*	24.3	31.8

Top row: the % difference between ACSC-corrected and non-corrected data is higher for controls than for phantom data. An in-depth investigation (rows 2 and 3) reveals that this discrepancy is due to SC: in fact, while the AC lead to a similar increase compared to the NC values (~16%) for both phantom and controls, the SC has a higher impact for the human controls

In Table 5, the percentages of the total counts in the satellite windows relatively to the photopeak are tabulated for phantom and human data for each of the gamma cameras included in this study; the percentage differences between these two groups are listed in the last two columns. Besides the variation between manufacturers in collimation performance, these results demonstrate a considerable difference between phantom and human data for all cameras ﻿in the upper window SC_u_, which substantiates the hypothesis that the contribution of extra-brain activity is relevant.

The conclusive proof of this deduction comes from the comparison of the SBR from NC and ACSC data for phantom and controls (for the Southampton method) in Table 6. The percentage difference for (ACSC-IRNC) is significantly higher in controls (48%) than in phantom studies (40%) (*p* < 0.001, two-sample with equal variance *t* test performed on the % differences). Furthermore, by breaking down the contributions of the two corrections, the AC component produces a similar increase of the SBR (~16%), while the SC component is associated with a larger increase in the controls (32%) than in the phantom (24%). This indicates that SC, besides being the dominant correction, is indeed responsible for the difference between phantom and human studies.

## Discussion

The impact of the reconstruction method and of the quantification has been analysed for the EARL ENC-DAT database. Two simple reconstruction methods, FBP and OSEM without any corrections have been compared to the “gold standard” recommended by EARL, OSEM with AC, and SC corrections. The salient characteristics of these databases—the mean age-corrected SBR value and its natural variability—are summarised in Table 1.

Contrary to expectations, the difference between the FBP and IRNC databases, although small was found to be significant, even after phantom calibration (Table 1). This may be an indication that OSEM has not reached full convergence. On this point, the work by Seret and co-workers [12] recommended a much higher number of iterations (24 iterations with 8 subsets) for state-of-the-art quantitative OSEM algorithms that incorporates attenuation, scatter, resolution recovery, and noise suppression. Even accounting for the fact that resolution recovery per se requires an increase in iterations, this number is comparatively higher than the one used for the ENC-DAT database [2]. Such high number of iterations, however, would not be feasible with the ENC-DAT databases for two fundamental reasons. Firstly, the ENC-DAT data have been acquired following a clinical protocol (in terms of injected activity and acquisition time), which results in a number of total counts (~2 million) two order of magnitude lower than those obtained in their phantom work (~100 million). Secondly, the basic OSEM used in the ENC-DAT reconstructions does not have the noise-suppression capabilities that come with resolution modelling reconstructions to counteract the steady increase of noise with the number of iterations. Furthermore, the observation that calibration cannot fully resolve the difference between FBP and IRNC databases seems to suggest possible differences in the OSEM performance when dealing with both phantom and human data. OSEM convergence has been observed to be variable across different phantom filling ratios and, in particular, to struggle at the higher SBR values associated with low count concentrations in the background compartment used in this study [2], due to its non-negativity constraint. Human studies, on the other hand, are expected to have relatively similar background concentrations irrespectively of their striatal uptake, and should therefore be similarly affected by the non-negativity constraint across all SBR values; variability in the background concentration and striatal uptake, however, would be expected across different cameras and collimators. Convergence with iterative reconstruction in clinical studies is complex and deserves further investigation, but this is outside the remit of this work.

The ACSC reconstruction, recommended by ENC-DAT, brings a significant increase of the SBRs which, prior to phantom calibration, is of the order of 47% for both BRASS and Southampton databases (Fig. 3a, b and Fig. 4a, b, Table 2). This increase is in line with the expectations of AC boosting striatal counts relatively to the peripheral background, and SC improving the contrast between hot and cold regions.

The main imaging factor affecting quantification, however, remains the partial volume effect (PVE). Its magnitude can be estimated from the percentage difference of the BRASS and Southampton databases before the introduction of phantom calibration. The systematic difference of these two methods, in fact, is ultimately due to their different approach to the PVE (Fig. 1). While BRASS, based on direct measure of counts concentration from tight striatal ROIs, is susceptible to partial volume losses, the Southampton method, based on the Specific Uptake Size Index (SUSI) approach, is able to overcome them [5]. Furthermore, the respective choices for the reference region—the occipital cortex for BRASS and the whole brain without the striatal VOI for the Southampton method—will also contribute to their different outcome [13]. Their difference can be fully appreciated by comparing their respective pre-calibration graphs in Figs. 3 and 4a, b, where the BRASS SBRs range is much lower than the Southampton one. The magnitude of this difference, of the order of 96% (Table 3), is a clear indication of how partial volume losses outweigh by far the 47% under-estimation related to attenuation and scatter/septal penetration (Table 2). This is in line with published literature [14].

Phantom calibration brings significant changes to the databases, with a large increase of the SBR values particularly for BRASS. The aim of the calibration is, in fact, not only the harmonization of the differences in performance between different camera models, but also the recovery of the “true” SBR values. In a sense, the calibration can be thought as having three “recovery components”, dealing with the AC, SC, and PVE degradations respectively. Depending on the database used, these components may be “turned on” or “off”, and can act in combination or in isolation. For example, the AC and SC recovery components will always be “turned on” when calibrating FBP or IRNC databases, but “off” for ACSC ones; the PVE component will be always “on” for BRASS databases but “off” for Southampton ones. As for their relative magnitudes, PVE recovery is the dominant component, hence responsible for the larger changes of the calibrated SBRs, followed by SC and finally by AC (Table 6).

Accordingly, the calibration corrections needed to recover the true SBR values are much larger for BRASS than for the Southampton method, as summarised in Table 4. For all BRASS databases, the outcome of the calibration is in fact dominated by the PVE recovery, which leads to a 67% increase of the ACSC database (Fig. 3b, d). Calibration of the FBP/IRNC databases, which incorporates the additional AC and SC recoveries, leads to an increase of the order of 100% (Fig. 3a–c). In the case of the Southampton method, the calibration has to deal, in principle, with AC and SC compensations only when not applied during reconstruction; consequently, it is expected to have a significant impact on the FBP/IRNC databases but not on the ACSC one. This is confirmed by the results of Fig. 4 and Table 4, which reveal a significant increase of ~31% when comparing FBP/IRNC pre- and post-calibration (Fig. 4a, c), but no significant effect on the ACSC database (Fig. 4b, d, *p* = 0.44).

When considering the inter-subject variability of the databases, as expressed by the standard deviation of the age-corrected SBRs (Table 1), it is noticeable how the calibration tends to increase the variability, particularly for BRASS. At first, this may appear disconcerting given the expectation that phantom calibration is aimed to harmonize camera performance and therefore to reduce variability. One possible explanation is that calibration, in recovering the “true” values, is actually restoring the true natural variability, which was somehow “lost” or “masked” by SPECT degradations. For BRASS ACSC, therefore, calibration will bring a pronounced increase in data variability, as its primary effect is to unmask and compensate for the differences in resolution performance across the various gamma cameras. For the Southampton databases, on the other hand, the data-variability is more consistent, as the confounding factor of PVE is inherently eliminated at source. In particular, the Southampton ACSC is the only case where the calibration brings a minor (and not significant) decrease of variability, likely to represent the result of harmonisation of residual equipment-related differences.

In principle, if full recovery was possible, phantom calibration should lead to equivalence of all databases, no matter what reconstructions or quantification methods was used. In reality, despite becoming much closer to each other, the calibrated databases remain significantly different. The success of calibration is ultimately determined by the ability of the phantom study to reproduce the clinical situation. The striatal phantom, however, is an approximate representation of a human study, due to a combination of factors such as the shape of the striatal vessels somewhat different from the human anatomy, the uniformity of the “non-specific” background that ignores the ventricular space void of activity and, above all, the lack of scatter and septal penetration of the radiation emitted from distant parts of the body. This is particularly relevant for ^123^I because of the presence of low-abundance highly-penetrating emissions, as demonstrated by the comparison of phantom and human results at both raw data level (projection counts, Table 5) and quantification level (SBR, Table 6). In Table 5, differences in scatter and septal penetration between phantom and human data are negligible for the SC_l_ window (they oscillates around 0) but show a marked increase in humans for the SC_u_ window (last two columns). As expected, the stopping capability of medium energy collimators (Philips IRIX) is noticeably superior to the low energy ones used in all other cameras (columns 3 and 5). Interestingly, a marked difference in collimator performance across manufacturers is also evident.

The fact that the phantom is not fully representative of a human study suggests that calibration can be though as a “first order”, camera-specific, compensation. Subject-specific “second order” effects, associated with the individual anatomy and tracer binding, can only be corrected by subject-data driven approaches. Consequently, calibration alone is not sufficient to fully resolve databases differences nor can ensure full recovery of the “true” SBR.

This would give a new insight in explaining the results in Table 1. The significant differences, between the non-corrected (FBP and IRNC) databases and the ACSC one, still present after calibration, can be explained as “second order effects” related to the fact that scatter and septal penetration correction are performed on individual basis for the latter, but as generic camera-dependent compensations for the former. Furthermore, the observation that the differences between the calibrated FBP, IRNC and ACSC databases are relatively smaller for BRASS compared to Southampton, can be explained as a direct reflection of the dominance of partial volume recovery in the BRASS calibration for all three databases, the magnitude of which would mask the more subtle second-order effects associated with their different approaches, generic or patient-driven, to scatter compensation.

Similarly, the fact that the differences between the BRASS and Southampton databases remain significantly large after calibration, ACSC mean values of 6.4 and 9.0, respectively, underlies the phantom capability to compensate for PVE at a first-order level only. The proposition that the Southampton ACSC mean SBR of 9.0 could be a close representation of the “true” value is supported by the work by Soret et al [15], which reports a mean of 8.6 in patients suffering from Alzheimer’s disease (this neurodegenerative disease is not characterized by loss of striatal dopamine transporters) obtained by applying, beside ACSC, an individualised MRI-driven partial volume correction [16] to a counts concentration “BRASS-like” calculation of the SBR.

Of the compensation methods for scatter and septal penetration, a known disadvantage of TEW compared to alternatives such as transmission-dependent convolution subtraction (TDCS) [17] is the increase of Poissonian noise in the projections data. However, being patient-driven, TEW has the advantage of being able to take into account the individuality of the tracer distribution in the whole body and to correct for its effect on the brain image, an individuality which is ignored by the pre-determined camera-specific factors used by TDSC. The observed reduction, ~10%, in inter-subject variability recently reported for the ENC-DAT ACSC database when using TDCS as opposed to TEW [13] could therefore be explained as natural variability which is missed by this methodology.

The ENC-DAT database has been acquired without the CT component because it was not available on most of the participating cameras. Access to SPECT/CT systems in clinical practice would provide CT-derived attenuation maps which, besides delivering a more accurate attenuation correction, could also be incorporated in iterative reconstructions for driving scatter corrections based on Monte Carlo simulation algorithms [18]. In these cases, however, the adoption of the ENC-DAT database in clinical use would require further validations, to assess the extent of the differences of the SBR values obtained with the different attenuation and scatter correction methods. The latter, again, would not be able to account for the extra-body activity and, therefore, could lead to SBRs significantly different from those obtained with TEW.

Although outside the scope of this study, it is worth mentioning that there are further confounding aspects encountered in routine clinical investigations which have an effect on resolution and SBR quantification. Tremor-related patient movements and radii of rotation larger than that standard 15 cm used for the ENC-DAT database, sometimes necessary to accommodate for patient anatomy or claustrophobia, may lead to significant reductions of the SBR [19–21]. In particular, the radius-dependence of the SBR should be considered as a “second order” effect which the phantom calibration cannot correct for, and whose severity depends on both reconstruction and quantification methods. While the Southampton method was found to be not affected by it, the use of morphological VOIs led to a loss of approximately 3% per cm additional radius [21], which should be taken in consideration when using the ENC-DAT database in clinical practice.

Ultimately, the results of this study are representative of the on-going struggle between robustness and accuracy in SPECT imaging. The determination of an accurate SBR would require attenuation, scatter and partial volume correction to be subject-data driven, with phantom calibration having the function of removing residual camera-related variability. On the other hand, phantom-driven compensations will produce an “index” less dependent on the reconstruction method at the expense of accuracy and loss of individual variability. The results of the present study do not allow conclusions about the impact of reconstruction and quantification methods on the diagnostic utility of the specific binding ratio; its clinical relevance in the context of the six databases here considered has been investigated in the companion paper by Dickson et al [8].

## Conclusions

The ENC-DAT normal database is dependent on reconstruction and quantification methods; hence its clinical use requires consistency in image processing and analysis. Phantom calibration, by providing a “first-order” harmonisation correction, is able to resolve the largest part of the differences between the various methodologies, but cannot establish full equivalence of different databases. In particular, FBP and IRNC databases remain significantly different from the recommended ACSC one, mainly because the scatter and septal penetration contribution from distant parts of the body is not accounted for in the phantom calibration. Caution must therefore be exercised when comparing data from different centres: awareness of the processing methodology is paramount together with the recognition that the SBR may represent an “index” rather that a “true” value.

## Abbreviations

AC: Attenuation correction
ACSC: OSEM with attenuation and scatter and septal penetration corrections
CoV: Coefficient of variation
EARL: EANM Research Ltd
ENC-DAT: European Normal Control Database of DaTSCAN
FBP: Filtered back projection without any correction
IRAC: Iterative reconstruction (OSEM) with attenuation correction
IRNC: Iterative reconstruction (OSEM) without any correction
OSEM: Ordered subsets expectation maximisation
PVE: Partial volume effect
SBR: Specific binding ratio
SD: Standard deviation
SC: Scatter and septal penetration correction
SE: Standard error of the regression
TDCS: Transmission-dependent convolution subtraction
TEW: Triple energy window
